# Comment on “Commercial landscape of noninvasive prenatal testing in the United States”

**DOI:** 10.1002/pd.4185

**Published:** 2013-09-02

**Authors:** Matthew Rabinowitz

**Affiliations:** 1 Natera Inc.San Carlos, CA, USA

We read with interest the recent review in Prenatal Diagnosis entitled “Commercial landscape of noninvasive prenatal testing in the United States” by Ashwin Agarwal *et al*. 1 We appreciate the attention afforded to the business and intellectual property landscape given by noninvasive prenatal testing’s rapid evolution. However, Agarwal *et al*. misrepresented the current commercial turnaround time for Natera’s single nucleotide polymorphism based Panorama™ test. This is an important parameter that affects clinical implementation, and as such, we feel it is important to report the most up-to-date information. We thus wish to communicate the preliminary results of analysis of the most recent 10000 commercially-processed samples.

Specifically, Panorama now reports a turnaround time of 5 to 10 business days; >95% of the aforementioned commercial samples were processed in under 10 business days.
